# Bacteria–phage coevolution as a driver of ecological and evolutionary processes in microbial communities

**DOI:** 10.1111/1574-6976.12072

**Published:** 2014-03-27

**Authors:** Britt Koskella, Michael A Brockhurst

**Affiliations:** 1 BioSciences, University of ExeterCornwall, UK; 2 Department of Biology, University of YorkYork, UK

**Keywords:** bacteriophage, resistance, host–parasite, antagonistic, species interaction, infection

## Abstract

Bacteria–phage coevolution, the reciprocal evolution between bacterial hosts and the phages that infect them, is an important driver of ecological and evolutionary processes in microbial communities. There is growing evidence from both laboratory and natural populations that coevolution can maintain phenotypic and genetic diversity, increase the rate of bacterial and phage evolution and divergence, affect community structure, and shape the evolution of ecologically relevant bacterial traits. Although the study of bacteria–phage coevolution is still in its infancy, with open questions regarding the specificity of the interaction, the gene networks of coevolving partners, and the relative importance of the coevolving interaction in complex communities and environments, there have recently been major advancements in the field. In this review, we sum up our current understanding of bacteria–phage coevolution both in the laboratory and in nature, discuss recent findings on both the coevolutionary process itself and the impact of coevolution on bacterial phenotype, diversity and interactions with other species (particularly their eukaryotic hosts), and outline future directions for the field.

## Introduction

**Fig 1:**
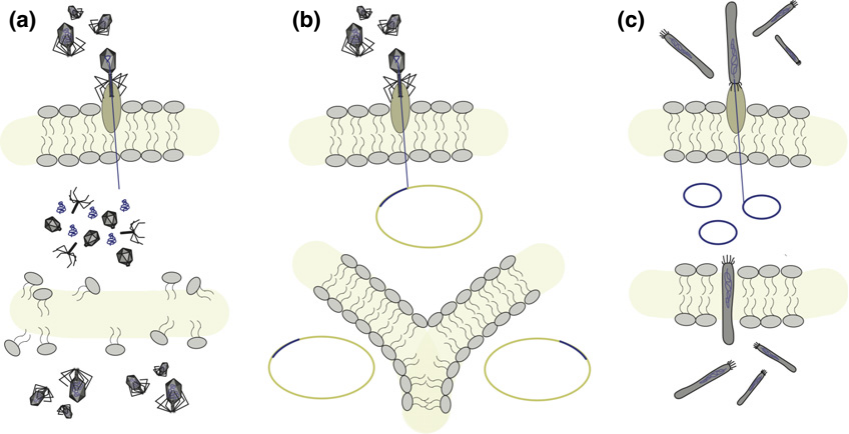
Diversity of phage reproductive strategies. (a) The ‘lytic’ phages replicate within their host cell and must burst the cell open to transmit to the next generation. These phages are therefore obligate killers of their hosts and are necessarily detrimental to host populations. (b) The ‘temperate’ phages (also referred to as ‘lysogenic’ phages or, once within the host genome, ‘prophages’) integrate into the host genome and reproduce along with the host cell. The integration of phage into the host genome can play a significant role in shaping bacterial phenotype and fitness (reviewed in Brüssow *et al*., 2004). (c) A relatively less common type of phage, the ‘filamentous’ phage, is able to reproduce without lysing the host cell and is continually secreted into the environment. These phages can also significantly alter bacterial phenotype, for example by encoding for toxins (Waldor & Mekalanos, 2012). Finally, the ‘cryptic prophages’ (not shown) are once temperate phages that have lost the ability to reproduce independently of their host (i.e. they can no longer enter the lytic cycle and transmit horizontally).

**Fig 2:**
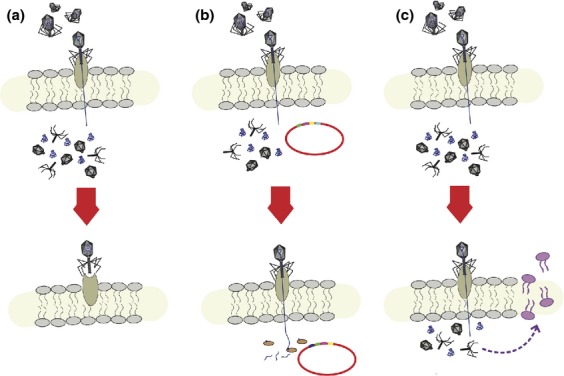
Illustration of bacterial resistance mechanisms against phages in the lytic cycle. There exist very few host-parasite systems for which the underlying mechanisms of infection and resistance are as well understood as bacteria–phage interactions, and yet new research continues to demonstrate the wide variety, complexity and sophistication of these coevolved systems (e.g. Høyland-Kroghsbo *et al.*, 2013; Seed *et al.*, 2013). Bacteria have evolved a number of defense mechanisms against invading phages, including those that (a) prevent phage adsorption, (b) degrade phage DNA inside the cell and/or block replication, and (c) initiate cell death upon infection. First, (a) bacteria can lose or alter the target receptor of phages, can produce an extracellular matrix of polysaccharides that blocks phage attachment, or can produce competitive inhibitors that bind to the phage attachment site. Phages can counter-adapt by altering their tail fiber attachment sites, by producing enzymes to degrade the matrix, or by changing the receptors to which they attach. (b) After successful phage attachment, bacteria can still prevent infection using a restriction-modification system, whereby recognized phage DNA is degraded by restriction enzymes, or through the CRISPR-Cas system, which has been identified in over 40% of bacteria and 90% of archaea (reviewed in Westra *et al.*, 2012). (c) Finally, many bacterial species have mechanisms that lead to cell death, for example by degrading the cell wall, upon successful phage infection, thereby protecting neighboring cells from further infection. However, phages have been known to evolve mechanisms to evade even this level of defense by mimicking the host's defensive system (Blower *et al.*, 2012).

Bacteria–phage interactions are central to the ecology and evolution of microbial communities. Phages are known to alter competition among bacterial strains/species (e.g. Bohannan & Lenski, 2000a, b; Joo *et al.*, 2006; Koskella *et al.*, 2012), maintain bacterial diversity (e.g. Buckling & Rainey, 2002a, b; Rodriguez-Valera *et al.*, 2009), and mediate horizontal gene transfer among bacteria (e.g. Kidambi *et al.*, 1994; Canchaya *et al.*, 2003). Phages have evolved a diversity of life histories and transmission strategies to exploit prokaryotic host cells for their own reproduction (Fig. 1); phages with a temperate lifestyle and filamentous phages are capable of forming long-term associations with bacterial cells, through lysogeny and pseudolysogeny, while phages with an exclusively lytic lifestyle are obligate killers of their host, requiring lysis to transmit to the next host cell (reviewed in Clokie *et al.*, 2011; Abedon, 2012). Phages are abundant in natural ecosystems, often outnumbering coexisting bacteria (Thomas *et al.*, 2011; Williamson *et al.*, 2013; Engelhardt *et al.*, 2014), and can impose significant mortality on their bacterial hosts (Faruque *et al.*, 2005; Allen *et al.*, 2010; Shapiro *et al.*, 2010; Clokie *et al.*, 2011). Bacteria can readily evolve resistance to phage attack by *de novo* mutation and have a diverse arsenal of other mechanisms with which to defend themselves against phage infections (Fig. 2). Given the abundance of phages and potential impact of phage-mediated selection on bacterial populations, there is growing interest among microbial ecologists to understand the coevolutionary processes underlying interactions between bacteria and lytic phages. The mechanisms of phage infectivity and bacterial resistance to phages have been reviewed extensively elsewhere (Nechaev & Severinov, 2008; Hyman & Abedon, 2010; Labrie *et al.*, 2010; Westra *et al.*, 2012; Molineux & Panja, 2013; Samson *et al.*, 2013; Young, 2013), as has the potential impact of temperate phages on the fitness and phenotype of their bacterial hosts, for example through horizontal transfer of functional genes (Brüssow *et al*., 2004; Stern & Sorek, 2011; Varani *et al.*, 2013). Therefore, we focus this review on the feedback between microbial ecology and bacteria–phage coevolution.

Coevolution is defined as the process of reciprocal adaptation and counter-adaptation between ecologically interacting species (Janzen, 1980). Bacteria–phage systems have led the way as model systems for understanding mechanisms of infection and genome evolution, but have lagged dramatically behind other host-parasite systems in the integration of ecology and coevolution into a working understanding of the interaction. This is unfortunate given the insights gained from other systems to the role that coevolution plays in shaping genome evolution (Hosokawa *et al.*, 2006; Kerstes *et al.*, 2012), driving divergence among populations (Benkman, 1999), maintaining diversity within populations (Koskella & Lively, 2009; Morran *et al.*, 2011), and even ecosystem-level processes (Stouffer & Bascompte, 2011). The evidence from bacteria–phage systems thus far suggests that populations are highly dynamic over time and rapidly (co)evolving. For example, a study following changes in the human gut virome over two and a half years demonstrates both high persistence of phage lineages through time and rapid substitution rates, particularly among those phages known to be lytic (Minot *et al.*, 2013). Analysis of microbiota from 124 human hosts used the integration of phage DNA into the clustered regularly interspaced short palindromic repeats (CRISPR) regions of the bacterial genome to infer past selection pressure from phages and was able to demonstrate both a clear role for phages in shaping the microbiome and a potential reservoir of shared phages among individuals (Stern *et al.*, 2012). Similar dynamical changes have been observed for bacterial and phage communities in the ocean, where associations seem to fluctuate over the course of days and yet are relatively stable over longer time scales (Needham *et al.*, 2013). Indeed, it is now widely accepted that phages act to maintain microbial diversity at many levels and across ecosystems: within genomes (Banfield & Young, 2009; Avrani *et al.*, 2011); within populations (Brockhurst *et al.*, 2004; Benmayor *et al.*, 2008); among populations (Buckling & Rainey, 2002a Morgan *et al.*, 2005); and within communities (Bohannan & Lenski, 2000b; Brockhurst *et al.*, 2006; Duerkop *et al.*, 2012).

In this review, we summarize the empirical evidence for coevolution between bacteria and phages arising both from laboratory experimentation and from studies of natural communities. We report that (1) sustained coevolution is a common albeit not a universal outcome of copropagation of bacteria and phage and can be detected in natural bacteria–phage communities; (2) the dynamics and outcomes of bacteria–phage coevolution are often contingent upon environmental conditions in predictable ways; and (3) the process of coevolution can impact bacteria and phage diversity at all levels (from genomes to communities) and significantly affects bacterial phenotype. We focus primarily on interactions between bacteria and lytic phages, rather than temperate phages or interactions among archaea and their viruses (we refer readers to a previous review of archaea–virus interactions; Snyder & Young, 2011), because the former have proven much more amenable to laboratory testing than the latter and have therefore been the focus of the majority of experimental coevolution studies (for a general overview of this approach, see Brockhurst & Koskella, 2013). In addition, we use the terms ‘host’ and ‘parasite’ throughout the review to refer to an interaction from which one player benefits (the parasite) to the other's detriment (the host), for example via reduced fecundity or death. As obligate killers of their host cells, whereby they must burst the cell open to release infectious virons into the environment and infect future hosts, lytic phages can usefully be considered parasites (or parasitoids) for the sake of applying predictions from theory and comparing the systems to other antagonistic interactions.

## Coevolution in the laboratory

### Potentially endless cycles of defense and counter-defense

Early laboratory studies of bacteria–phage interactions, largely using *Escherichia coli* B as a host to one of various tailed-phages [*Caudovirales*, including the myoviruses T2 (Paynter & Bungay, 1969) & T4 (Horne, 1970), the podovirus T7 (Chao *et al.*, 1977), and the siphoviruses, including T1 (Schrag & Mittler, 1996), T5 (Lenski & Levin, 1985), and λ-vir (Spanakis & Horne, 1987)], suggested that bacteria–phage coevolution was limited to between 0.5 and 1.5 cycles of reciprocal evolutionary change (where a cycle is the evolution of bacterial resistance followed by the evolution of phage infectivity, often termed a host-range mutant). In all cases, coevolution appeared to cease after the emergence of a resistant bacterial genotype that the phage could not evolve to overcome (reviewed in Dennehy, 2012). Resistance in these studies generally derived from *de novo* mutations causing modifications to the structure of bacterial surface molecules targeted by phages, which prevented phage attachment (Lenski & Levin, 1985). Similar results of constrained coevolution have been documented for cyanobacteria and cyanophages (Cowlishaw & Mrsa, 1975; Cannon *et al.*, 1976; Barnet *et al.*, 1981), *Vibrio cholerae* and vibriophages (Wei *et al.*, 2010, 2011), and various other marine bacteria (Waterbury & Valois, 1993; Middelboe *et al.*, 2001). Lenski and Levin concluded that bacteria–phage coevolution was fundamentally constrained by an asymmetry of evolutionary potential between bacteria and phages (Lenski & Levin, 1985). They argued that this asymmetry arises because many mutational routes to receptor modification or loss can achieve resistance, while phage infectivity requires the evolution of specific binding to the modified version of the existing receptor or to an entirely different receptor, which is likely to be subject to greater mutational constraint.

Despite the logic and likely generality of this mutational asymmetry, evidence from a range of other bacteria–phage interactions suggests that prolonged bouts of recurrent coevolutionary cycles can and indeed do occur in laboratory culture. The most intensively studied bacteria–phage interaction, in terms of the pattern and process of its coevolution, is that between *Pseudomonas fluorescens* SBW25 and the T7-like podovirus, Φ2 (Buckling & Rainey, 2002b; Brockhurst *et al.*, 2007a). When propagated by batch culture in rich media, these species undergo persistent coevolution during long-term experiments (*c*. 450 bacterial generations; Buckling & Rainey, 2002a, b; Hall *et al.*, 2011a). The coevolutionary interaction of *P. fluorescens* and Φ2 is comprised of two distinct phases (Hall *et al.*, 2011b). During the first 200–250 bacterial generations, coevolution proceeds through a series of recurrent, reciprocal selective sweeps, whereby directional selection drives the fixation of new bacterial resistance mutations followed by new phage mutations restoring infectivity (Hall *et al.*, 2011b). Consequently, bacteria and phages evolve to become, respectively, more broadly resistant (i.e. can resist a greater number of phage genotypes) and more broadly infectious (i.e. can infect a greater number of bacterial genotypes) over time (Buckling & Rainey, 2002a, b). This mode of coevolution is often termed an ‘arms race’ due to the escalation of defense and counter-defense traits by both species (Dawkins & Krebs, 1979; Woolhouse *et al.*, 2002; Gandon *et al.*, 2008). Qualitatively similar arms-race coevolution has also been observed in chemostat cultures of *E. coli* O157:H7 with the T4-like myovirus, PP01 (Mizoguchi *et al.*, 2003), of *Cellulophaga baltica* and phi-S (Middelboe *et al.*, 2009), and of the cyanobacterium *Synechococcus* with the myovirus, RIM8 (Marston *et al.*, 2012).

After *c*. 250 bacterial generations, the rate of arms-race coevolution between *P. fluorescens* and Φ2 decelerates, such that both bacterial resistance range and phage infectivity range reach their asymptotes. This occurs due to progressively weaker responses to directional selection over time, due primarily to costs of generalism in both the bacteria and the phages (Hall *et al.*, 2011b). In bacteria, resistance mutations altering cell-surface lipopolysaccharide molecules (Scanlan *et al.*, 2013) are associated with high pleiotropic costs due to impaired function of these molecules, which reduces fitness (Brockhurst *et al.*, 2004; Buckling *et al.*, 2006). For the phages, mutations conferring broader infectivity range often occur in genes encoding host-attachment proteins (Paterson *et al.*, 2010; Scanlan *et al.*, 2011). The precise nature of the associated costs of generalism is poorly characterized but generalist phages appear to suffer impaired growth rates (Poullain *et al.*, 2008). Thus, arms-race coevolution eventually gives way to sustained oscillations of bacterial and phage genotypes with different resistance and infectivity specificities, respectively (Hall *et al.*, 2011b). These oscillations are driven by negative frequency-dependent selection. By this process, phages evolve to infect common bacterial genotypes, giving an advantage to rare bacterial resistance alleles, which rise in frequency, and so on, indefinitely. This mode of coevolution is termed fluctuating selection dynamics (Gandon *et al.*, 2008).

Intriguingly, there is evidence of mutational asymmetry between *P. fluorescens* and phage Φ2 of the kind hypothesized by Lenski and Levin to impose a fundamental constraint upon bacteria–phage coevolution (Lenski & Levin, 1985). Specifically, whereas bacteria can readily evolve broad resistance ranges via single spontaneous mutations, phage evolution of broad infectivity ranges during coevolution seems to require the stepwise acquisition of multiple mutations and is relatively more constrained (Hall *et al.*, 2011a, b). The implication, therefore, is that despite the mutational asymmetry, extensive bacteria–phage coevolution is possible, even in the absence of environmental heterogeneity or biotic complexity, although further data from alternate systems are required before any broad generalizations can be made. Why then was coevolution so constrained in early studies using *E. coli* B? One possibility is that *E. coli* B has a long history of laboratory cultivation (Daegelen *et al.*, 2009), whereas the bacterial strains capable of extensive coevolution were much more recently isolated from the environment [*P. fluorescens* SBW25 from a sugar beet leaf (Bailey *et al.*, 1995), *E. coli* O157:H7 from a diarrheal disease outbreak (Mizoguchi *et al.*, 2003), and *Synechoccus* spp. from the ocean (Marston *et al.*, 2012)]. Indeed, *E. coli* B has multiple known defects in its lipopolysaccharide (Yoon *et al.*, 2012), a key attachment site for many tailed phages (e.g. T4, T7, and T2; Lenski & Levin, 1985), which may limit its scope for coevolution with certain phages. Similarly, the tailed-phages employed in early studies were also highly laboratory-adapted following many growth cycles on *E. coli* B prior to the advent of cryogenic storage (Demerec & Fano, 1945). This inadvertent selection for specialization on a particular host receptor may have constrained the subsequent evolutionary potential of the phages.

A fascinating exception comes from recent experiments between *E. coli* B and λ-vir where an extensive arms race is only observed following the evolution of a ‘key innovation’ by the phage to bind to a new host receptor against which it has no history of prior adaptation (OmpF instead of LamB; Meyer *et al.*, 2012). This, along with the evidence from the other interactions described above, suggests that extensive arms races may be a common feature of newly constituted interactions between tailed-phages and bacteria (or their previously untargeted receptors). Other demographic factors may also counteract the mutational asymmetry constraint. For instance, phages typically have shorter generation times and larger population sizes than their bacterial hosts, both of which are thought to enhance evolutionary potential in host-parasite systems (Gandon & Michalakis, 2002). Moreover, other forms of resistance, such as CRISPR-mediated resistance, require bacteria to specifically match the targeted phage (Barrangou *et al.*, 2007), which reverses the mutational asymmetry in favor of phages. Recent evidence suggests that phages can indeed readily evolve to escape recognition by CRISPR resistance (Levin *et al.*, 2013; Sun *et al.*, 2013). There is an urgent need for more empirical studies across a wide taxonomic range of bacteria–phage interactions to determine whether the patterns observed in studies so far are generalizable.

### Coevolution as a driver of diversity

Bacteria–phage coevolution plays an important role in shaping genotypic, phenotypic and community-level diversity (Weinbauer & Rassoulzadegan, 2004; Rodriguez-Valera *et al.*, 2009; Avrani *et al.*, 2011). Coevolution can promote high levels of within population diversity in terms of both bacterial resistance and phage infectivity phenotypes (Poullain *et al.*, 2008) and the underlying genotypes (Paterson *et al.*, 2010; Scanlan *et al.*, 2011). Coevolving lytic phages can increase diversity within bacterial populations by selecting for multiple modes of resistance (Brockhurst *et al.*, 2004, 2005; Benmayor *et al.*, 2008; Forde *et al.*, 2008), and both lytic (Bohannan & Lenski, 2000a, b) and temperate phages (Schwinghamer & Brockwell, 1978; Joo *et al.*, 2006) have been shown to alter apparent competition among bacterial strains. Moreover, the stochasticity of coevolutionary trajectories among populations can drive correlated phenotypic and genetic divergence (Paterson *et al.*, 2010). This has been observed both as variation in the dominant bacterial colony morphologies among populations (which presumably became linked to successful resistance mutations) (Buckling & Rainey, 2002a, b; Brockhurst *et al.*, 2004; Vogwill *et al.*, 2011) and divergence of bacterial resistance and phage infection specificities between populations giving rise to local adaptation (Buckling & Rainey, 2002a, b; Morgan *et al.*, 2005).

Recent work from an experimental *Streptococcus thermophiles*–phage system tracked metagenomic changes in the bacterial and phage populations after 1 week of coculturing (Sun *et al.*, 2013). The researchers examined acquisition of phage-related spacers in each of the two functional CRISPR loci within the bacterial genome and found that after 1 week, all host cells in the population had acquired at least one spacer that matched the phage genome, with a remarkably high level of CRISPR spacer diversity among individual bacteria within the population. The phage genome was also sequenced and three SNPs were documented, one of which was found in a proto-spacer region. Each of these mutations reached a frequency of over 88% in the phage population, suggesting that phage diversity was relatively minimal compared with that of the bacterial host population. Furthermore, a follow-on experiment shows that the common spacer in the bacterial population fluctuates over a 15-day period and also suggests strong selection pressure determining which regions of the phage genome become incorporated as spacers (Paez-Espino *et al.*, 2013). A similar asymmetry in bacterial and phage diversity was documented during a 6-month chemostat experiment using cyanobacteria and cyanophages (Marston *et al.*, 2012).

Within a community ecology context, phages hold potential to mediate competition among bacterial species, as has been discussed in great detail in light of the ‘kill the winner hypothesis’, in which population growth by otherwise dominant bacterial species is hampered by phage infection (Thingstad, 2000; Winter *et al.*, 2010). Results of several laboratory characterizing the ecological effects of phages on their hosts are consistent with a role for phages in maintaining bacterial diversity (Harcombe & Bull, 2005; Brockhurst *et al.*, 2006), and the introduction of narrow host range phages to replicate experimental, two species communities of marine bacteria significantly altered the biomass of the nonhost species (Middelboe *et al.*, 2003). However, how coevolution between bacteria and phages within a complex community setting might influence the interaction network and microbial diversity remain open questions (Beckett & Williams, 2013).

### Ecological contingency of bacteria–phage coevolution

Resistance of bacteria to lytic phages has been found to carry substantial fitness costs (Lenski, 1988; Bohannan *et al.*, 2002), including an increased cost of deleterious mutations (Buckling *et al.*, 2006), decreased ability to metabolize carbon (Middelboe *et al.*, 2009), altered competitive ability (Brockhurst *et al.*, 2005; Lennon *et al.*, 2007; Quance & Travisano, 2009), and increased susceptibility to other phages (Avrani *et al.*, 2011; Marston *et al.*, 2012). Ecological conditions can mediate the costs and benefits of resistance and infectivity for bacteria and phages, respectively (Messenger *et al.*, 1999; Jessup & Bohannan, 2008). Thus, the effects of several key ecological variables on bacteria–phage coevolution have been studied to identify conditions that promote or constrain coevolutionary dynamics. Increasing dispersal, or mixing, within populations can increase contact rates between bacteria and phages, and can be achieved by as simple a manipulation as periodically shaking the culture vessel (e.g. experiments comparing static and shaken cultures of *P. fluorescens* and Φ2; Brockhurst *et al.*, 2003). Population mixing enhances phage transmission, thereby increasing the benefit of resistance to bacteria. This effect strengthens selection for the evolution of resistance in bacteria, which correspondingly strengthens the selection upon phages to restore infectivity, and, overall, accelerates arms-race coevolution (Brockhurst *et al.*, 2003). Dispersal at larger spatial scales, that is, between populations or patches within a metapopulation, can also accelerate coevolution; however, this effect is critically dependent upon the rate of dispersal. Low to intermediate rates of dispersal (< 1% per generation approximately) of bacteria and phages, or bacteria or phages alone, can accelerate coevolution by increasing the supply of beneficial genetic variation, which enhances the response of the dispersing species to reciprocal selection (Lively 2009; Brockhurst *et al.*, 2007b; Morgan *et al.*, 2007; Vogwill *et al.*, 2008). In contrast, high rates of dispersal (> 10% per generation approximately) act to homogenize genetic variation and synchronize coevolutionary dynamics across populations (Vogwill *et al.*, 2009a; Vogwill *et al.*, 2011), thereby diminishing the benefits of dispersal and leading to rates of arms-race coevolution similar to those observed in isolated populations (Morgan *et al.*, 2007; Vogwill *et al.*, 2008).

Increasing the rate of supply of resources (i.e. the concentration of carbon substrates) to bacteria reduces the cost of resistance mutations and accelerates the rate at which such mutations arise and invade populations (Bohannan & Lenski, 1997; Harrison *et al.*, 2013). Bacterial population densities also increase with resource supply, which can lead to elevated bacteria–phage contact rates, potentially enhancing the benefits of resistance. Combined, these population genetic and ecological effects of increased resource supply both act to intensify reciprocal selection and enhance the bacterial response to phage-mediated selection, accelerating coevolutionary dynamics (Lopez-Pascua & Buckling, 2008). As a result of differences in mutational supply, the strength of selection and the relative costs of resistance mutations, different levels of resource supply select for qualitatively different bacterial resistance mutations, and correspondingly, phage infectivity mutations (Forde *et al.*, 2008; Lopez-Pascua *et al.*, 2012). This causes greater divergence among populations of contrasting resource supply levels than is observed among populations with equivalent resource supply levels, causing stronger patterns of local adaptation (Lopez-Pascua *et al.*, 2012). Therefore, resource supply affects not only the *rate* of bacteria–phage coevolution, but also its *trajectory*.

Several studies using the *P. fluorescens*-Φ2 and *E. coli*-T7 interactions have considered the effects of spatial ecological heterogeneity on bacteria–phage coevolutionary dynamics (Forde *et al.*, 2004, 2007; Vogwill *et al.*, 2009a, b; Lopez-Pascua *et al.*, 2010). In particular, these experiments have manipulated dispersal of bacteria and phages between populations propagated under different ecological conditions that vary in the intensity of coevolution [e.g. between populations with high vs. low resource supply (Forde *et al.*, 2004; Lopez-Pascua *et al.*, 2010) or high vs. low rates of population mixing (Vogwill *et al.*, 2009a)]. In general, these studies reveal that, given dispersal, the rate of coevolution at the ‘landscape level’ (i.e. across all connected populations) is set by the fastest coevolving population, which acts as a coevolutionary pacemaker (Vogwill *et al.*, 2009b). The effects of temporal heterogeneity on bacteria–phage coevolution have been less extensively studied. Recent experiments with *P. fluorescens-*Φ2 manipulated the frequency of fluctuations in resource supply by propagating populations in environments that alternated between high and low resource supply at different rates. Rapidly fluctuating environments (e.g. alternating every *c*. 7.5–15 bacterial generations) constrained arms-race coevolution relative to constant environments, whereas slowly fluctuating environments (e.g. alternating every *c*. 30 bacterial generations) did not. This occurred because selective sweeps of bacterial resistance mutations, which were only ever observed under high resource supply, required *c*. 25 bacterial generations to occur and were therefore impeded by rapid fluctuations in resource supply (Harrison *et al.*, 2013).

While most experimental coevolution has employed pairs of species, bacteria–phage interactions often occur embedded within a diverse microbial community. Relatively few experimental coevolution studies have attempted to scale up community complexity beyond pairwise interactions. However, recent experiments suggest that addition of multiple exploiters of bacteria, such as other phages or protist predators, may limit the ability of bacteria to evolve defense against a focal phage species. For example, the evolution of phage resistance in the plant-pathogenic bacterium, *P. syringae,* was found to differ depending on the heterogeneity of the phage population. Those bacterial populations that were coevolving with multiple phage genotypes were able to evolve resistance as readily as populations coevolving with single phage genotypes, but the former paid a greater cost for such resistance (Koskella *et al.*, 2012). Similarly, *P. aeruginosa* strains evolved in the presence of two phages were found to have decreased growth rate and motility relative to those evolved with a single phage but increased production of siderophores (Hosseinidoust *et al.*, 2013). The addition of a protist predator, *Tetrahymena thermophila*, to experimental populations of *P. fluorescens* and Φ2 impeded coevolution between the bacteria and phage, but favored the maintenance of coexisting resistance phenotypes specialized against one or other of these natural enemies (Friman & Buckling, 2012).

## Coevolution in the wild

Given the overwhelming data from experimental laboratory studies for the ecological contingency of bacteria–phage coevolution, the key question becomes whether what we know about these interactions in the laboratory can be directly translated to make predictions about what happens in natural microbial communities. There are a number of reasons to think that this might not be straightforward; the additional abiotic and biotic selection pressures, variation in resources, competition among species, and vastly differing population sizes and migration rates are all likely to alter the trajectory of bacteria–phage coevolution relative to what is observed in simple microcosm experiments. Although the underlying process should remain the same, it becomes a daunting task to predict which specific features of the natural world might be most influential in altering the trajectory of bacteria–phage coevolution. The wealth of literature describing bacterial and phage diversity in nature (reviewed in Weinbauer, 2004; Breitbart & Rohwer, 2005; Clokie *et al.*, 2011) is suggestive of on-going coevolutionary dynamics, but the role of coevolution relative to other factors such as dispersal or selection by the abiotic environment in shaping this diversity has rarely been examined.

### Seminatural environment microcosm studies

One elegant approach is to bridge the gap between purely experimental and purely observational (and thus correlational) studies by running experiments under seminatural conditions. Gomez and Buckling have applied this approach by measuring coevolution between bacteria and phages in soil microcosms that are either sterile prior to introduction of the target bacteria and phage clones or that contain a natural community of microorganisms (Gómez & Buckling, 2011). In this way, they have successfully demonstrated that coevolution between marked strains of *P. fluorescens* and phage Φ2 in soil is more in line with fluctuating selection than arms-race dynamics, as neither the bacteria nor phage become increasingly resistant/infective over time. This is in stark contrast to previous laboratory studies using the same system but run in nutrient-rich media broth (Brockhurst *et al.*, 2003, 2007a, b). One explanation for the observed difference is that resistance is more costly in the soil environment than in the broth environment and therefore that resistance to previous phage types is lost in favor of specific resistance to contemporary phage. This increased cost of resistance would both constrain the continual arms-race selection toward increased resistance/infectivity and lead to fluctuating dynamics as new resistances are gained and old resistances are lost. Indeed, Gómez and Buckling (2011) go on to show that resistant bacterial strains evolved in broth were no less fit than their sensitive ancestor, while those which had evolved resistance in soil had a 36% reduction in fitness. Intriguingly, they found no substantial difference in coevolutionary outcome between those bacteria and phages coevolving in the presence vs. absence of the natural microbial community. This result suggests that phage-mediated selection may be strong enough to override selection arising from interspecific competition. Furthermore, the work demonstrates that experiments with added complexity have a great deal to offer for elucidating both which components of natural systems are important in predicting the outcome of coevolution and those that might be less important than predicted. More recently, this same seminatural system has been used to demonstrate that coevolving phages do not select for increased bacterial mutation rate (Gómez & Buckling, 2013), as had been previously observed from studies *in vitro* (Pal *et al.*, 2007). Similarly, seminatural mesocosm studies of phytoplankton and their associated viral populations have demonstrated long-term genotypic stability over time in a natural fjord (Martínez *et al*., 2007) and arms-race dynamics during bloom development (Vardi *et al.*, 2012).

### Evidence for bacteria–phage coevolution in nature

Given the multiple competing selection pressures and the fact that bacteria–phage encounter rates in nature are likely to be dramatically different than those observed in liquid microcosms, it is unclear exactly how important of an evolutionary force phages are in shaping natural microbial populations. However, there is building evidence from across a number of bacteria–phage systems that coevolution is occurring and having significant ecological impact in nature. The first critical step in demonstrating a role for phages in driving bacterial evolution has been quantifying both their prevalence and host range in nature. One way to indirectly test for the impact of lytic phages in shaping microbial diversity is to remove them from natural populations/communities and determine whether there is a corresponding change in genotypic or community composition. For example, the depletion of viral particles from seawater led to a marked change in the relative abundances of marine bacterioplankton (Bouvier & Del Giorgio, 2007), and the manipulation of the presence of viral and protist predators in seminatural communities of bacterioplankton found both increased bacterial richness in the presence of phages and a complex interaction between phages and protists on bacterial mortality that ranged from synergistic to antagonistic (Weinbauer *et al.*, 2007).

Thanks to the power of metagenomics, the true extent of phage prevalence is being uncovered, and it is now clear that phages are ubiquitous across natural systems and can account for the turnover of *c*. 20% of the living biomass in the sea (Suttle, 2007; Clokie *et al.*, 2011). For example, a newly discovered phage (HMO-2011) capable of infecting the most abundant lineage of marine bacteria, the SAR116 clade, can account for up to 25% of all viral genome reads in the ocean (Kang *et al.*, 2013). Sequence-based estimates of phage abundance are likely to be significantly higher than estimates based on quantification of infective phage particles [for example, abundance estimates of cyanophages infecting cyanobacteria in the open ocean were found to be two orders of magnitude lower than that of their hosts (Sullivan *et al.*, 2003)], but do not allow for a complete understanding of the infection network within natural populations and communities. In particular, sequence-based approaches do not allow for estimates of infection prevalence, as it is typically not possible to determine the host range of a given phage. For example, evidence from *Salmonella* bacteria and their associated phages on a dairy farm suggests both great diversity of phages, and a high density of multiple specialist phage types, each capable of infecting common bacterial strains (Moreno Switt *et al.*, 2013). A statistical analysis of large phage host range datasets has recently been introduced and applied to bacteria–phage interaction networks from soil (Poisot *et al.*, 2013), the ocean (Flores *et al.*, 2013) and a meta-analysis of 38 laboratory-tested networks (Flores *et al.*, 2011). Overall, these data support the idea that phages in nature span the continuum from specialist to generalist, resulting in what is known as a ‘nested’ structure (reviewed in Weitz *et al.*, 2013; Martiny *et al.*, 2014). The next key steps in the study of bacteria–phage coevolution can be broken down into those data supporting: (1) dynamical change in bacterial and phage communities over time; (2) phage adaptation to bacterial populations; (3) bacterial response to phage-mediated selection; and (4) finally, reciprocal change of phage and bacterial communities over time. Each of which we review below.

There are a number of sophisticated studies that support fluctuations in bacterial populations and corresponding phage populations over time (Waterbury & Valois, 1993; Mathias *et al.*, 1995; Cochran & Paul, 1998; Marston & Sallee, 2003; Faruque *et al.*, 2005). Phage abundance in a bioreactor was observed to change frequently and was correlated with changes in the corresponding bacterial taxonomic group (Shapiro *et al.*, 2010). Cyanobacteria and their associated cyanophages were also found to be highly dynamic. By tracking mutational change in a phage tail sheath gene over both 5-year and 1-day sampling periods, Kimura and coauthors were able to demonstrate both short- and long-term oscillations of the five major cyanophage genotypes, suggestive of frequency-dependent dynamics (Kimura *et al.*, 2013). In concert with previous findings from the same system showing highly diverse CRISPR sequences in the host population (Kuno *et al.*, 2012), this work reveals strong associations and rapid evolution of the bacterial and phage populations. Similarly, dynamic change was reported for a population of *Sphingomonas* sp. and its lytic phage in a freshwater lake in Northern Germany over the course of 3 months (Jost & Wiese, 2013). The population dynamics observed will of course vary both according to the timeframe and the resolution of analysis. For example, phage and bacterial communities from four aquaculture or solar saltern environments were monitored over time at both a course and fine-grain taxonomic scale. At the scale of the species, both microbial and viral communities remained stable, with the top microbial and viral taxa persisting over periods ranging from a single day to over a year (Rodriguez-Brito *et al.*, 2010). At the strain level, however, the authors observed continuous variation in the abundance of both viral and bacterial genotypes.

**Fig 3:**
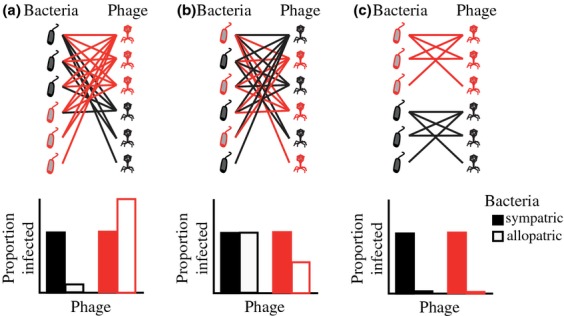
An illustration of phage local adaptation across two populations. In the first case, (a) the bacteria–phage network across the two populations is nested, and the population containing phages with the broadest host range (in red) also contains the most resistant bacteria. This structure, which is loosely based on directional, arms-race selection, does not result in an overall pattern of local adaptation. In the second case, (b) the network remains nested, but phage host range and bacterial resistance are not correlated with population. Again, this structure does not result in an overall pattern of local adaptation. In the final case, (c) phages from one population are only infective to bacteria from the same population, although the network structure within each population remains nested. Unlike the others, this structure would lead to an overall pattern of phage local adaptation. Overall, we suggest that if phage-mediated selection typically leads to an increase in generalized resistance to phage attack, phages from one population should be most infective to hosts from populations under relaxed phage-mediated selection and least infective to those from populations under relatively strong phage-mediated selection, regardless of sympatry. Phage local adaptation therefore suggests both that hosts are unable to evolve resistance rapidly enough to escape their local phages and that phage infectivity/bacterial resistance is relatively specific.

There is empirical data to support that phages in nature are locally adapted to their bacterial hosts (Fig. 3; Vos *et al.*, 2009; Gómez & Buckling, 2011; Koskella *et al.*, 2011). This is in contrast to laboratory studies where bacterial local adaptation (i.e. where the phages do particularly poorly on their coevolving hosts relative to others) is often found (Buckling & Rainey, 2002a, b; Morgan *et al.*, 2005; Vogwill *et al.*, 2010). In soil from a flood plain near Oxford, phages collected from only 1 cm apart were found to be more infective to bacterial hosts (mainly *Stenotrophomonas*) from those same samples than they were to neighboring bacterial isolates (Vos *et al.*, 2009). This study confirms that bacteria–phage interactions can be very local indeed and suggest that phages are adapting rapidly to bacterial strains in close proximity. In contrast, phage populations collected from the leaves of horse chestnut trees were found to be just as infective to bacterial hosts from other leaves within the same tree canopy as those from their own leaf (Koskella *et al.*, 2011). In this case, phage local adaptation was only apparent when comparing phage infectivity on bacterial hosts from neighboring trees, and this was true both for the whole bacterial community and also for those isolates that were identified as *P. syringae*. These patterns observed in terrestrial systems may not translate to aquatic and marine communities, where evidence from co-existing populations of cyanobacteria and phages has demonstrated that, despite a high abundance of phage, most circulating host types are in fact resistant to local phages (Waterbury & Valois, 1993).

Insight into the scale of bacteria–phage coevolutionary dynamics can also be gleaned from studies that attempt to match the CRISPR spacers in bacterial genomes to phage DNA found from the same location. For example, in this way, researchers have examined datasets from the human microbiome project and found segregation of CRISPR sequence according to the body-site from which the sample was taken (Rho *et al.*, 2012). Furthermore, by comparing the CRISPR spacer sequences across body sites and among individuals, they were able to demonstrate that, while resampling of the same site from the same individual recovered many of the same spacers, different individuals had almost no spacers in common. A similar approach was taken to investigate adaptation of *Candidatus Accumulibacter phosphatis* across geographically separated bioreactors (Kunin *et al.*, 2008). In this case, despite evidence for high dispersal among the sites and relatively little divergence among bacterial genotypes overall, the authors were able to demonstrate rapid divergence of CRISPR sequences which correlated with local phage-mediated selection. Furthermore, the two regions of the bacterial genome suggested to be evolving under phage-mediated selection, the CRISPR elements and the EPS gene clusters, were also the regions found to be most different among closely related strains of *S. thermophilus* isolated from yogurt manufactured in France and in the United Kingdom (Bolotin *et al.*, 2004). Together, these genomic and phenotypic studies suggest that phages are capable of rapidly adapting to their bacterial hosts and that natural bacterial host populations will respond to this selection by evolving increased resistance.

Direct evidence for a response to phage-mediated selection in nature has proven somewhat more difficult to obtain. A recent study from microbial communities living within the leaves of horse chestnut trees utilized a ‘time-shift’ approach (Gandon *et al.*, 2008; Gaba & Ebert, 2009) to determine whether bacteria were more resistant to phages from relatively earlier in the season compared with those from later in the season (Koskella, 2013). Indeed, bacterial isolates were found to be most resistant to phages from the prior month and least resistant to phages from 1 month in the future, suggesting a rapid response to phage-mediated selection that could be explained by mutational change or immigration of new and resistant strains and species. Similarly, phages were found to be most infective to bacteria from earlier in the season and least infective to those from the future, to which they had not yet adapted.

### Coevolving gene networks

The measure of bacteria–phage coevolution in nature has been hampered by our lack of understanding of the interaction networks of the host and parasite. As mentioned above, this understanding is increasing with sophisticated statistical models and meta-analyses of large datasets (Weitz *et al.*, 2013). Specifically, knowing whether phages commonly have narrow host ranges, infecting only a single or multiple genotypes of a single host species, or broad host ranges, infecting multiple species or even species spanning multiple genera, is a prerequisite for elucidating the impact that phages will have on their host population and community and vice versa (Fig. 3; Sullivan *et al.*, 2003). To illustrate this point, imagine that a single phage is capable of infecting two different bacterial species in its local environment. These two bacteria may evolve resistance via different mechanisms, or by incorporating different phage-derived spacers, but they may also evolve resistance via a shared mechanism or spacer region. Under the former scenario, the phage may also diversify, creating the potential for two new pairwise coevolving bacteria–phage lineages. However, under the latter scenario, the phage may well adapt to overcome the shared mechanism and the process of coevolution is pairwise at the gene level, but not the species level. Thus, the need for resolution of infection networks at the gene level, rather than species level, is pressing.

There are reasons to think that phage specificity (i.e. narrow host range), as opposed to generalism (broad host range), is the rule rather than the exception in nature (reviewed in Hyman & Abedon, 2010; Koskella & Meaden, 2013; Martiny *et al.*, 2014). Although our understanding of what constitutes a narrow or broad host range is necessarily hampered by the reference panel against which specificity is measured, the infection networks that have been analyzed to date illustrate a continuum between narrow and broad host range, with most phages infecting some but not all of the panel of bacteria they are confronted with, suggesting some constraint upon host range even among the most broadly infectious phages. Constraints on host range could arise due to fitness trade-offs (Duffy *et al.*, 2006), differences between hosts in terms of overall quality (Heineman *et al.*, 2008), or intracellular defenses against successfully adsorbed phages (Sieber & Gudelj, 2014). Regardless of the exact degree of specificity of the interaction, it is clear that many phages infect more than one bacterial strain or species and that many bacteria can act as host to a number of different phages in the environment (Flores *et al.*, 2011). Thus, bacteria–phage coevolution is unlikely to be purely pairwise and we might instead think of bacterial and phage communities as coevolving gene networks, whereby particular phage genes are under selection by corresponding bacterial genes, regardless of which host genome they might be found. The breakdown of simple pairwise coevolution in bacteria–phage systems is further exacerbated by the process of horizontal transfer of genes among bacterial genomes (Smillie *et al.*, 2011), much of which is phage-mediated (Canchaya *et al.*, 2003), and the potential for recombination among phage genomes during coinfection (Benbow *et al.*, 1974; Riede, 1986; Shcherbakov *et al.*, 1992; Worobey & Holmes, 1999; Lin *et al.*, 2012). Evidence from cyanobacterial populations even supports the movement of mutations conferring resistance to phage among bacterial genomes via HGT of a hypervariable genomic island (Avrani *et al.*, 2011). Importantly, recombination among phage genomes has been shown to alter host range (Riede, 1986; Lin *et al.*, 2012), and early theory set out to describe phage evolution as a process occurring at the modular level, where interchangeable genetic elements were recombined to create the optimal phenotype at any given time (Botstein, 1980). The complexity of untangling interactions among genes from interactions among organisms has been highlighted in a recent review of the microbiome (Boon *et al.*, 2014), and many of the same complexities exist when exploring bacteria–phage networks in nature.

## Future directions

Throughout the review, we have attempted to highlight the progress that has been made in our understanding of coevolution between bacteria and phages, as well as to emphasize that our understanding is still rapidly developing. Even our comprehension of bacterial resistance mechanisms and phage infectivity is continually improving. The relatively recent discovery of CRISPRs as a defense against phage (Barrangou *et al.*, 2007; Andersson & Banfield, 2008) has already been expanded to include anti-CRISPR counter-measures by phages (Bondy-Denomy *et al.*, 2012) and to show that phages can also carry a CRISPR-cas system to target a chromosomal island of the bacterial host (Seed *et al.*, 2013). Furthermore, the importance of bacterial suicide upon phage infection has recently been demonstrated in *E. coli* and was found to be a low cost strategy for reducing the population-wide impact of phage (Refardt *et al.*, 2013) that is favored in spatially structured environments (Berngruber *et al.*, 2013). However, the coevolutionary implications of these new mechanisms have not yet been explored, and this avenue is ripe for empirical testing using an experimental coevolution approach and for examination of natural patterns in the field.

Another key advance of the field will be incorporation of both theoretical and empirical examination of coevolution between bacteria and temperate, as well as filamentous phages. There are a number of reasons to expect the coevolutionary process to differ for these interactions relative to those with lytic phage. Primarily, many of these phages confer a strong fitness benefit to their hosts and thus will act more as mutualists than parasites. This can shift dynamics from parasite-mediated negative frequency-dependent selection (where hosts are constantly evolving to defend themselves against the common parasite) to positive frequency-dependent selection, where for example, carrying the lysogenic phage confers resistance to the same phage in the lytic form and therefore the benefit of being a lysogen increases with the frequency of other lysogens. Similarly, filamentous phages can increase the fitness of their hosts through toxin production and increased pathogenicity, as has been found for *V. cholerae*, the causative agent of cholera (Waldor & Mekalanos, 1996). Both filamentous and temperate phage systems have proven amenable to *in vitro* experimentation, but, to our knowledge, have not been used to test for coevolution. One-sided experimental evolution of the filamentous phage f1 demonstrated increased virulence (in terms of larger impact on population density) when horizontal transmission among hosts was increased relative to vertical transmission within a dividing bacterial lineage (Messenger *et al.*, 1999), but it remains to be determined whether the bacterial population would respond by evolving increased resistance under these same conditions. One-sided experimental evolution of the lysogenic phage λ was also used to select for altered sensitivity and threshold for the switch from lysogenic to lytic phage life cycle (Refardt & Rainey, 2010).

Finally, further exploration of the similarities and differences between bacteriophages and other viruses will both help inform the utility of *in vitro* coevolution studies as a basis for building predictions for other virus–cell interactions, and uncover any unique adaptations of phages to their bacterial hosts. For example, examination of the archaeon, *Sulfolobus islandicus,* and its associated viral parasites isolated from hot springs suggests a clear biogeographic structure, such that viral genomes were found to be specifically associated with each local host population (Held & Whitaker, 2009). This system has led the way in uncovering the parallel role of CRISPR systems in archaea–virus interactions and reinforces evidence from bacteria–phage systems that demonstrate a role for viruses in maintaining host diversity (Held *et al.*, 2010). In addition, an examination of temporal dynamics of archaea–virus interactions in a hypersaline lake suggests ample change over the course of both months and years, indicating similar timescales and mechanisms for these interactions as observed with bacteria–phage systems (Emerson *et al.*, 2013). The other similarities between archaea–virus and bacteria–phage interaction have been reviewed elsewhere (Snyder & Young, 2011). Finally, it remains to be seen whether our increasing understanding of bacteria–phage coevolution will prove useful in studies of eukaryote-virus interactions, but at the least, each body of work could help shape the questions addressed in and techniques utilized by the other (Brockhurst *et al.*, 2007a; Sharp & Simmonds, 2011).
